# Clinical Impact of Psychopathy on Bipolar Disorder

**DOI:** 10.3390/medicina57020165

**Published:** 2021-02-12

**Authors:** Giuseppina Calabrò, Antonio Francesco Musolino, Andrea Adolfo Filippo, Renato de Filippis, Elvira Anna Carbone, Marianna Rania, Matteo Aloi, Valentina Pugliese, Cristina Segura-Garcia

**Affiliations:** 1 Department of Health Sciences, University Magna Graecia of Catanzaro, 88100 Catanzaro, Italy; giusy878@gmail.com (G.C.); antoniof.musolino@gmail.com (A.F.M.); andreafilippo369@gmail.com (A.A.F.); defilippisrenato@gmail.com (R.d.F.); elvira.carbone@libero.it (E.A.C.); marianna.rania@hotmail.it (M.R.); matteo.aloi@hotmail.it (M.A.); valen.pugliese@libero.it (V.P.); 2 Department of Medical and Surgical Sciences, University Magna Graecia of Catanzaro, 88100 Catanzaro, Italy

**Keywords:** psychopathy, bipolar disorder, comorbidity, empathy, impulsiveness, prevalence

## Abstract

*Background and Objectives*. Bipolar disorder (BD) is associated with a significant burden due to affective symptoms and behavioral manifestations, but also cognitive and functional impairment. Comorbidity with other psychiatric conditions, including personality disorders, is frequent. The comorbidity with psychopathy deserves special consideration given that both disorders share some clinical characteristics, such as grandiosity, risky behavior or poor insight, among others, that can worsen the outcome of BD. Therefore, this study aimed to evaluate the prevalence of psychopathy in a sample of clinically stabilized patients with BD and its impact on the severity of BD. *Materials and Methods*. A sample of 111 patients with BD (38 type I and 73 type II) was studied. The Hamilton Depression Rating Scale (HAM-D) and the Young Mania Rating Scale (YMRS) served to assess the severity of BD. Psychopathy was measured by means of the Psychopathic Personality Inventory-Revised (PPI-R). Patients were divided into three groups according to the severity of psychopathy (Group 1: no psychopathy; Group 2: “psychopathic” trait; Group 3: clinical psychopathy). Other measures regarded impulsiveness (Barratt Impulsiveness Scale-11, BIS-11) and empathy (Empathy Quotient, EQ). Comparisons of mania, depression, impulsivity and empathy scores were run with MANOVA considering psychopathy and diagnosis as independent variables. *Results*. The prevalence of psychopathy was 5.4%. A significant association between the level of psychopathy and YMRS, attentional/cognitive impulsivity and motor impulsivity scores emerged. No interaction between psychopathy and BD diagnosis was found. Post hoc analysis demonstrated significantly higher YMRS scores in Group 3 than in Group 1; that is, patients with psychopathy have more manic symptoms. *Conclusion*. Psychopathy seems quite frequent among patients with BD. The association of psychopathy with BD results in higher impulsivity and manic symptoms. In light of this, psychopathy should be investigated when assessing patients with BD, regardless of the comorbidity of BD with other personality disorders.

## 1. Introduction

Bipolar disorder (BD) is a recurrent chronic disorder characterized by mood and energy fluctuations [1] that affects about 1.5% of the population [2]. It involves episodes of mania or hypomania, with hyperactivity and uninhibited behavior and interspersed episodes of depression with profound anhedonia [3]; in any case, extreme clinical and therapeutic heterogeneity is observed, both intra-individual and inter-individual.

Given its chronic nature, BD is associated with a significant burden mainly due to the cognitive and functional impairment that results in a persistent alteration in the trajectory and quality of life [4,5]. Several conditions can compromise health and quality of life in BD [6]; among them, comorbid psychiatric disorders worsen the course of illness and outcome [7] especially if an underlying Personality Disorder (PD) is present. The prevalence of a comorbid PD in BD is about 41% [8] and several studies reported increased severity, suicidal risk and drug treatment resistance due to this association [9].

An increased risk of arrest and incarceration was also observed in BD [6,10,11] regardless of impulsivity related to comorbidity with borderline personality disorder (BPD) or antisocial personality disorder (ASPD) [12], which alone does not explain the most serious crimes. Although higher impulsivity can be present during the manic episode, impulsivity itself should not be considered a BD trait since it is not observed during euthymia [13,14].

The comorbidity between BD and PD was investigated [8,12,14] but, to our knowledge, no studies clearly evaluated the relationship between BD and psychopathy, a construct that might be considered as a contributor to the severity of BD regardless of the underlying PD.

Psychopathy is a personality disorder that includes emotional, interpersonal, and behavioral components like deceptiveness, grandiosity, impulsiveness, boldness, fearlessness, antisociality, and a lack of empathy [15,16]. “Psychopaths” were described as “social predators who charm, manipulate, and ruthlessly plow their way through life... completely lacking in conscience and feelings for others, they selfishly take what they want and do as they please, violating social norms and expectations without the slightest sense of guilt or regret” [17]. Theories on psychopathic personality disorder have a rich historical tradition, and strong empirical base, but despite this, according to the conceptualization of the Diagnostic and Statistical Manual of Mental Disorders-5 (DSM-5), ASPD and psychopathy are not considered distinct entities [18]. The new theoretical model of psychopathy identifies four dimensions that reflect disturbances in interpersonal and emotional functioning, as well as impulse control and social functioning [19]. It is therefore a pathological personality style that is interpersonally deceptive, affectively cold, behaviorally reckless, and often overtly antisocial [20].

Approximately 1% of psychiatric patients are “psychopathic”, as assessed by the Hare Psychopathy Checklist, Revised (PCL-R) [21] and BD and psychopathy are likely to share some core symptoms. For this reason, we hypothesize that the comorbidity of these two severe disorders could, in theory, account for a more impaired psychopathology of BD.

Therefore, the aim of our study is to evaluate the prevalence of psychopathy in a sample of clinically stabilized patients with BD and its impact on the psychopathology of BD.

## 2. Materials and Methods

### 2.1. Participants

Trained psychiatrists recruited participants at the Outpatients Unit of Psychiatry of the University Hospital “Mater Domini” of Catanzaro between 2017 and 2019. The inclusion criteria were patients aged 18–65 years old with a diagnosis of Bipolar Disorder type I or II according to DSM-5 (American Psychiatric Association, 2013) [22] confirmed through the Structured Clinical Interview for DSM-5 (SCID-5-CV) [23]. Exclusion criteria were diagnosis of dementia, intellectual disability or other neurological disorders associated with psychiatric symptoms; substance abuse; conditions that did not allow the completion of the assessment, such as language problems, dyslexia or poor knowledge of Italian language.

The final sample consisted of 111 patients, 38 diagnosed with type I bipolar disorder and 73 with type II bipolar disorder. Researchers informed each potential candidate about the methods and purposes, the anonymity of data collected and the non-obligation to participate. All subjects provided written informed consent to participation according to the local ethics committee guidelines. The study was done in accordance with the latest version of the Declaration of Helsinki (World Medical Association W (2018) WMA Declaration of Helsinki–Ethical principles for medical research involving human subjects) and approved by the Ethics Committee of “Comitato Etico Sezione Area Centro della Regione Calabria” (protocol code 109; 27 April 2017).

### 2.2. Procedures

Collected data included socio-demographics (i.e., age, sex, education level, civil status, occupation), clinical information, medical and psychiatric comorbidities and psychopharmacological therapy in place.

For the clinical assessment, researchers administered the Young Mania Rating Scale (YMRS) [24] and Hamilton Depression Rating Scale (HAM-D) [25] to evaluate the absence or presence, and severity if present, of maniacal or depressive symptoms. Afterwards, patients answered the Barratt Impulsiveness Rating Scale (BIS-11) [26], the Empathy Quotient (EQ) [27,28] and the Psychopathic Personality Inventory-Revised (PPI-R) [29]. The BIS-11 is composed of 30 items with three oblique factors: attentional/cognitive impulsivity (BIS_AI), measuring tolerance for cognitive complexity and persistence; motor impulsivity (BIS_MI), measuring the tendency to act on the spur of the moment; and non-planning impulsivity (BIS_NPI), measuring the lack of sense of the future. Items are rated from 1 (absent) to 4 (most extreme) [30]. The EQ has 60 items with 40 questions tapping empathy and 20 filler items. Responses are given on 4-point Likert scale and scores can range from 0 to 80. The PPI-R measures psychopathic personality traits through 154 4-point Likert-type items (i.e., false, mostly false, mostly true, or true) that produce 8 factors: Machiavellian Egocentricity (ME), Rebellious Nonconformity (RN), Blame Externalization (BE), Carefree Nonplanfulness (CN), Social Influence (SOI), Fearlessness (F), Stress Immunity (STI), and Coldheartedness (C). The measure yields a total score and two higher-order orthogonal factors weakly correlated: Fearless Dominance (FD = SOI + F + STI), which captures emotional and interpersonal aspects of psychopathic personality and Self-Centered Impulsivity (SCI = ME + RN + BE + CN), which captures impulsive traits and irresponsible lifestyle [31]. The average score in the general population is 50 ± 10 [29] and PPI-R ≥ 65 corresponds to clinically significant psychopathy [29]. In our sample, the PPI-R score served to divide patients into three groups according to the severity of psychopathy (Group 1: no psychopathy; Group 2: “psychopathic” trait; Group 3: clinical psychopathy). Further, the validity factor of PPI-R and specifically the inconsistent responding (IR) 15 was controlled for: the test is inconsistent and invalid if IR15 > 17 [29].

### 2.3. Statistical Analysis

IBM SPSS Statistics (version 26.0) software was used for database construction and statistical analysis. Data are presented as means, standard deviations (SD), frequencies and percentages (%). YMRS, HDRS, and BIS scores were not normally distributed according to the Kolmogorov–Smirnov test, and therefore they were transformed into log-scores. According to PPI-R scores [29], the sample was divided into three groups for further comparisons: Group 1 (PPI-R < 60: no psychopathy), Group 2 (60 ≥ PPI-R < 65: psychopathy trait) and Group 3 (PPI-R ≥ 65: clinical psychopathy). Prior to conducting two-way multivariate analysis of variance (MANOVA), a series of Pearson correlations were performed between all of the dependent variables in order to test the MANOVA assumption that the dependent variables would be correlated with each other in the moderate range (i.e., 0.20–0.60) [32]. The MANOVA was carried out with the PPI-R and diagnosis as independent variables, and with mania, depression, impulsivity and empathy scores as dependent variables. Tukey’s HSD post hoc test was run for significant results. Eta-squared (ƞ2) was used as a measure of the effect size of MANOVA, considering values of 0.01, 0.06 and 0.14 as indicating small, medium and large effects, respectively. Cohen’s d was used as a measure of effect size for post hoc comparisons, considering that values of 0.2, 0.5 and 0.8 respectively indicate low, medium and high effect size [33]. Bearing in mind the exploratory and naturalistic approach, *p* < 0.05 was considered to be significant. 

## 3. Results

The sample constituted 111 patients (48 males, 63 females). Table 1 shows the main characteristics of participants. Three groups emerged according to PPI-R scores: Group 1 (97 patients without psychopathy), Group 2 (8 patients with psychopathic trait) and Group 3 (6 patients with clinical psychopathy). Table 2 reassumes the mean scores of groups to tests.

A significant pattern of correlations was observed amongst most of the dependent variables, suggesting the appropriateness of a MANOVA. A multivariate ANOVA was then conducted. Findings revealed significant associations between the level of psychopathy and YMRS (F = 4.883; *p* = 0.01), BIS_AI (F = 3.426; *p* = 0.038) and BIS_MI (F = 4.164; *p* = 0.019) scores. There was no interaction effect of psychopathy by diagnosis (Wilks’ Lambda = 0.936; F = 0.950; *p* = 0.455).

Finally, a series of post-hoc analyses (Tukey’s HSD) examined individual mean difference comparisons across the three levels of psychopathy. The results revealed that patients from Group 3 (pathological psychopathy) had significantly higher YMRS mean scores than Group 1 (no psychopathy) (*p* < 0.05). That is, patients with psychopathy have more manic symptoms. The effect size, as estimated by Cohen’s d, was 0.2, which is a small effect according to Cohen’s guidelines (Table 3).

## 4. Discussion

The aims of our study were to evaluate the prevalence of psychopathy in a sample of clinically stabilized patients with BD and to examine the severity of BD psychopathology according to this comorbidity. To our knowledge this is the first study to evaluate the prevalence of psychopathy in a sample of BD patients by means of PPI-R. We hypothesized that the risky, violent or criminal behavior described as characteristic of patients with BD could be associated with psychopathy because manic episodes can more easily lead the patient to have criminal penalties, directly impacting quality of life, illegal conduct and poor treatment adherence [34]. The present study identified a subtype of patients deserving greater clinical attention from a psychopathological and probably therapeutic point of view. A fundamental problem in the treatment of patients with psychopathy is the lack of awareness of their condition. People with psychopathy rarely seek help; their poor insight, empathy and impulsiveness make them extremely dangerous for society [35].

Three groups emerged on the basis of increasing PPI-R scores. Group 3 collected participants with psychopathy and Group 2 consisted of patients with BD and psychopath trait. Compared with data from studies on the general population without a mental disorder [35], we found a very high prevalence of psychopathic disorder (5.4%) and psychopathic trait (7.2%) among patients with BD. On the other hand, this study confirmed the higher prevalence of psychopathic features among males [36]. The prevalence of psychopathy was similar in both BD subtypes, but patients with BD type II were over-represented in our sample, and this type is frequently comorbid with other PDs often worsening the outcome [14].

Fovet et al. described a high prevalence of BD among inmates [37] ranging between 2–7% [38,39]. Similarly, psychopathy, which from a conceptual point of view persists as separate from ASPD, has prevalence rates from 3% to 73% among prisoners, despite remarkable differences between countries [40,41,42,43]. On the other hand, regardless of the criminal behavior, psychopathy may be present in 1% of the general population [35]. All these reasons made us think about the interest of assessing the association between psychopathy and BD.

Those who tested positive to PPI-R never had any legal problems or were never incarcerated, supporting the evidence that psychopathy, either as a trait or a disorder, can be present in the general population [36]. This finding gains interest considering the relational and functional consequences of psychopathy in daily life. People with psychopathy have little awareness about their condition and do not consider mental health care necessary [36]; they do not perceive there is anything wrong with them and instead they think they have a great advantage over the rest of the community [17,44]. Therefore, this unawareness can only worsen if comorbidity with BD is added, especially during the manic/hypomanic episodes of the disorder [45]. If so, a delay in accessing care and/or an incorrect diagnosis are probable.

Group 3 showed higher levels of mania, depression and impulsivity in spite of clinical stabilization, which could be explained by means of a poorer therapeutic compliance due to the scarce awareness of both psychopathy and BD. Other authors also found that impulsivity tends to be high among subjects with psychopathy contributing to the severity of BD [12]; accordingly, in our study a more severe attentional and behavioral impulsivity were evident.

Current research failed to confirm the lower empathic capacity of individuals with psychopathy [36] as EQ scores were in line with normal levels of empathy quotient in all individuals regardless of the group they belonged to.

We wondered if more severe psychopathology was independent or related to comorbidity between BD and psychopathy. On the basis of our results, the psychopathic disorder seems to be the determining factor for a more severe psychopathology. Patients with BD, therefore, would once again suffer from a dysfunctional psychological impact on the course of their pathology, which will consequently be more severe and disabling [14]. Post-hoc analysis confirmed the influence of psychopathy on mania. Possibly, grandiosity or increased energy, psychopathic characteristics assessed by PPI-R, overlap with typical symptoms of an exaltation of mood. Therefore, the evaluation of psychopathy would be a useful parameter to define the prognosis of BD.

### Limitations

The first limits of this study are the small sample size and the consecutive recruitment of patients. This recruitment allows a more real-life condition (e.g., various ages of onset, various duration and severity of the disease, different psychopharmacological treatments) but limits the extension of results to the general population. Another limit is the cross-sectional design, which precludes the possibility of studying causality; a longitudinal study could have allowed us to understand whether psychopathy influences the therapeutic outcome of BD. Finally, no other personality traits or PD (e.g., BPD or ASPD) that could account for the higher severity of the “BD-psychopathy comorbidity” were evaluated. The third limit is the non-probability method of sampling that could hinder the generalization of the results obtained to the general population. For these reasons, future studies with random samples are needed in order to better understand the relationship between psychopathy and BD.

## 5. Conclusions

According to our results, psychopathy seems quite frequent among patients with BD and the comorbidity of BD and psychopathy appears associated with more severe impulsive and manic symptoms. Until now, clinicians could have interpreted them as residual symptoms or as indicators of resistance to treatment. The present results open up another possible explanation: symptoms of psychopathy become more evident after the clinical stabilization of BD. Thus, when assessing BD, one should consider the presence of psychopathy regardless of the association with another PD; this could be useful in planning more personalized and suitable psychopharmacological and psychotherapeutic interventions for the management of patients with BD for whom psychopathy could be more insidious and disabling.

## Figures and Tables

**Table 1:** Sample description.

		Total Sample		Group 1	Group 2	Group 3
		fr	%	%	%	%
Sex	male	48	57	43	38	67
	female	63	43	58	62	33
Age ^§^	current	44.3	12.7	12.6	9.1	16.5
	at onset	30.4	9.9	10	6.9	9.9
Bipolar subtype	BD I	38	34	37	25	0
	BD II	73	66	63	75	100
Education	Primary school	2	2	2	0	0
	High school	35	32	29	38	67
	College	48	43	45	25	33
	University	26	23	24	38	0
Civil status	single	43	39	41	38	0
	married	50	45	43	38	83
	separated	11	10	9	25	0
	divorced	5	5	5	0	0
	widow	2	2	1	0	17

^§^ Results presented as means and SD. BD: Bipolar disorder.

**Table 2:** Mean scores.

	Group 1		Group 2		Group 3		Total	
	Mean	SD	Mean	SD	Mean	SD	Mean	SD
YMRS	4.8	4.9	3.1	3.4	11.2	7.9	5.0	5.1
HAMD	6.4	5.9	10.0	10.1	11.2	7.1	7.0	6.4
BIS_AI	16.8	4.1	19.9	4.2	21.3	4.8	17.2	4.3
BIS_MI	21.6	5.1	24.8	7.9	25.2	4.4	22.0	5.4
BIS_NPI	30.6	5.4	31.5	4.6	32.3	9.0	30.7	5.6
BIS_Total	68.8	11.2	76.1	12.8	78.8	12.3	69.9	11.6
EQ	50.7	11.7	53.4	11.1	47.3	16.1	50.7	11.9

YMRS: Young Mania Rating Scale; HAMD: Hamilton Depression Rating Scale; BIS: Barratt Impulsiveness Scale; AI: Attentional Impulsivity; MI: Motor Impulsivity; NPI: Nonplanning Impulsivity; EQ: Empathy Quotient; SD: Standard Deviation.

**Table 3:** Results of two-way MANOVA.

	log YMRS			log HAMD			log BIS-AI			log BIS-MI			log BIS-NIP			log EQ		
	F	*p*	η^2^	F	*p*	η^2^	F	*p*	η^2^	F	*p*	η^2^	F	*p*	η^2^	F	*p*	η^2^
PPI-R	4.883	**0.01**	0.118	1.654	0.198		3.426	**0.038**	0.086	4.164	**0.019**	0.102	0.289	0.75		0.804	0.451	
Diagnosis	1.992	0.162		0.244	0.623		1.133	0.291		3.383	0.07		0.166	0.684		0.488	0.487	
PPI-R × Diagnosis	1.067	0.305		0.903	0.345		2.168	0.145		2.689	0.105		0.059	0.81		0.489	0.486	

PPI-R: Psychopathic Personality Inventory Revised; YMRS: Young Mania Rating Scale; HAMD: Hamilton Depression Rating Scale; BIS: Barratt Impulsiveness Scale; AI: Attentional Impulsivity; MI: Motor Impulsivity; NPI: Nonplanning Impulsivity; EQ: Empathy Quotient; SD: Standard Deviation. Significant results in bold. η^2^ only for significant results.

## Data Availability

Data available on request.
